# Circular RNA in Schizophrenia and Depression

**DOI:** 10.3389/fpsyt.2020.00392

**Published:** 2020-05-07

**Authors:** Zexuan Li, Sha Liu, Xinrong Li, Wentao Zhao, Jing Li, Yong Xu

**Affiliations:** ^1^ Shanxi Key Laboratory of Artificial Intelligence Assisted Diagnosis and Treatment for Mental Disorder, First Hospital of Shanxi Medical University, Taiyuan, China; ^2^ Department of Psychiatry, First Hospital/First Clinical Medical College of Shanxi Medical University, Taiyuan, China; ^3^ National Key Disciplines, Key Laboratory for Cellular Physiology of Ministry of Education, Department of Neurobiology, Shanxi Medical University, Taiyuan, China; ^4^ Department of Humanities and Social Science, Shanxi Medical University, Taiyuan, China

**Keywords:** schizophrenia (SZ), depression (DEP), circular RNA (circRNA), biological function, expression, epigenetic characteristics

## Abstract

Schizophrenia (SZ) and depression (DEP) are two common major psychiatric disorders that are associated with high risk of suicide. These disorders affect not only physical and mental health, but they also affect the social function of the individual. However, diagnoses of SZ and DEP are mainly based on symptomatic changes and the clinical experience of psychiatrists. These rather subjective measures can induce misdiagnoses and missed diagnoses. Therefore, it is necessary to further explore objective indexes for improving the early diagnoses and prognoses of SZ and DEP. Current research indicates that non-coding RNA (ncRNA) may play a role in the occurrence and development of SZ and DEP. Circular RNA (circRNA), as an important component of ncRNA, is associated with many biological functions, especially post-transcriptional regulation. Since circRNA is easily detected in peripheral blood and has a high degree of spatiotemporal tissue specificity and stability, these attributes provide us with a new idea to further explore the potential value for the diagnosis and treatment of SZ and DEP. Here, we summarize the classification, characteristics, and biological functions of circRNA and the most significant results of experimental studies, aiming to highlight the involvement of circRNA in SZ and DEP.

## Introduction

Among the great quantity molecular regulatory factors affecting gene expression, non-coding RNA (ncRNA) is known to be a class of important regulatory factors. NcRNA refers to a kind of RNAs that do not encode proteins, but can participate in genetic regulatory processes at multiple levels, including interactions with DNA, RNA, and proteins through a variety of mechanisms (1). Gene regulation of ncRNAs *via* epigenetics plays an important role in many major diseases, such as schizophrenia (SZ) and depression (DEP) (2–4). At present, SZ is considered to represent an umbrella of genetic diseases that are mainly characterized by a certain number of genetic-interaction networks and their differential clinical symptoms (5). For DEP, studies have proposed a pathogenic model that involves interactions among the environment, genetics, and epigenetics (6). Moreover, the high rate of comorbidity observed in psychiatric disorders indicates a large genetic correlation between each other, and the existence of shared molecular mechanisms involved in the occurrence and development of diseases (7). Sha Liu et al.’s genome-wide association study (GWAS) of seven psychiatric disorders (8), including SZ, DEP, bipolar disorder (BD), autism spectrum disorder (ASD), attention-deficit/hyperactivity disorder (ADHD), anxiety disorder (ANX), and neuroticism, also confirmed this. And they further found that chromosomes 5q14.3, 11q23.2, and 7p22.3 are the three genomic regions with the highest pleiotropic effects, suggesting that genetic factors have significant influence on SZ and DEP. Although few in-depth studies have been published on specific signaling pathways and networks regulated by ncRNAs in SZ and DEP, especially in terms of circular RNAs (circRNAs), it is possible that further elucidation of ncRNAs may be useful for better understanding the pathogenesis of SZ and DEP.

CircRNA, as an important type of ncRNA, forms an annular closed structure by undergoing reverse splicing, which was first discovered in plants in the 1970s (9). Since it was originally found only in a few transcriptional genes, circRNAs were considered to be a product of splicing errors from low-abundance transcriptional processes. However, this hypothesis has since been refuted. CircRNAs are widely present in human cells, some of which are expressed at as much as 10-fold higher levels compared to those of their linear isomer counterparts (10–12). CircRNA differs from linear RNA in that it is directly joined together to form a covalently closed structure without a 3′ tail and 5′ cap element (13–15). Due to the circular structure of circRNA, it is not easily degraded by RNA enzymes and also exhibits a high stability within cells (16, 17), enabling it to stably perform cellular functions over a longer period of time.

At present, the diagnostic criteria for SZ and DEP are still based solely on symptomology. Although diagnostic criteria—such as from the Diagnostic and Statistical Manual of Mental Disorders (Fifth Edition) (DSM-5) of American Psychiatric Association and the International Classification of Diseases (ICD-11)—are currently available, diagnoses and evaluations of treatment efficacies for SZ and DEP still primarily rely on clinical meetings with psychiatrists and lack objective physiological, biochemical, and pathological indicators. Hence, this more subjective diagnostic system can easily cause misdiagnoses and missed diagnoses. The above characteristics of circRNAs provide us with a novel approach to explore the biological basis for the occurrence and development of SZ and DEP. Therefore, the focus of this review is on circRNAs and their possible involvement in both SZ and DEP.

## Methods

We searched PubMed and GeenMedical for publications over the period of 1976 to 2019 and included the following the key phrases during searching: “circular RNA and neuropsychiatric disorders” “circular RNA and schizophrenia” “circular RNA and depression” and “circular RNA and biological function”. Only papers in English were used in the preparation of this review. The references obtained were screened by the authors to determine which ones would be included for discussion in the present review.

## Overview of circRNAs

### Classifications and Looping Mechanisms of circRNAs

**Figure 1:**
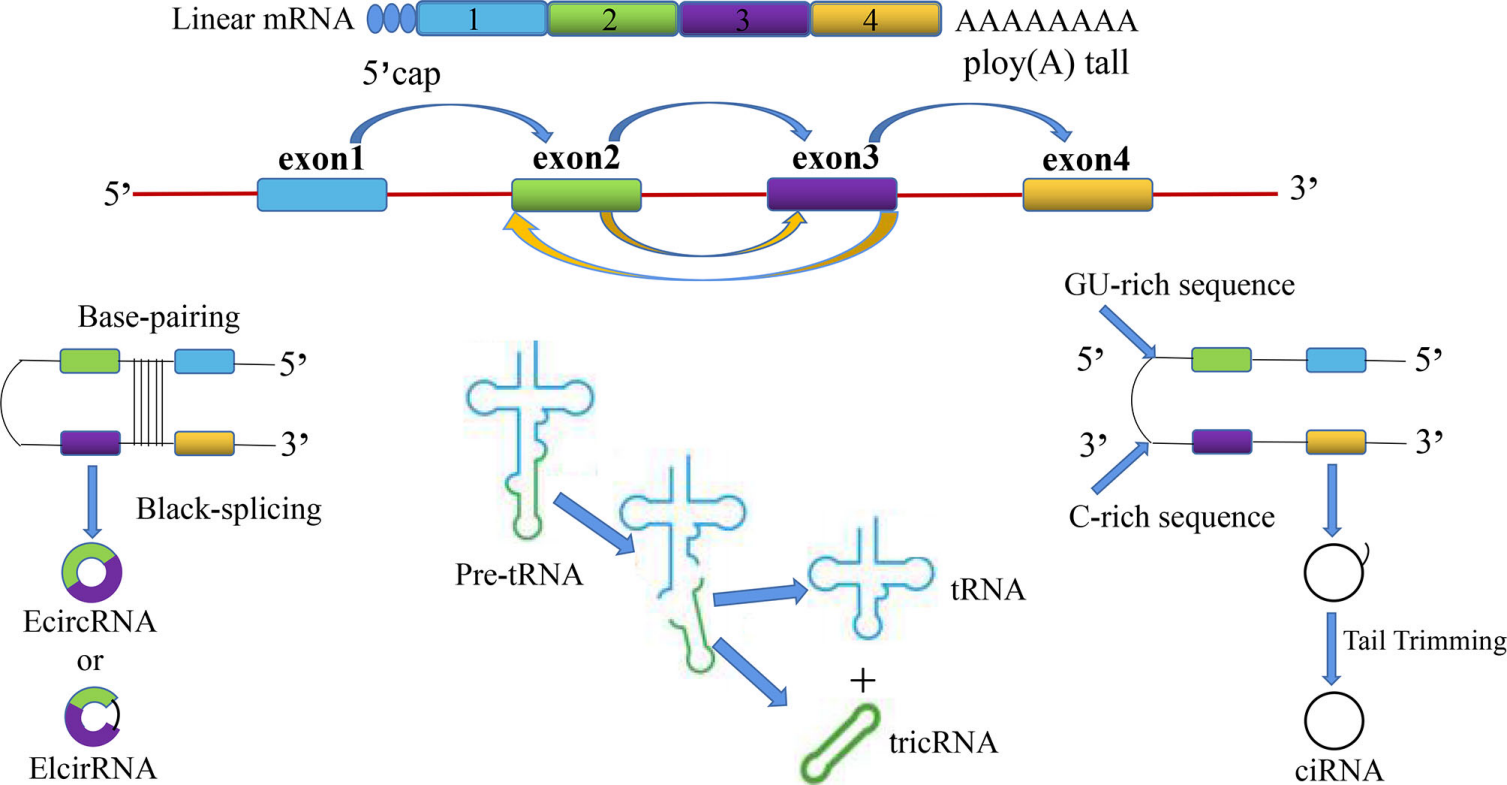
The overview diagram shows four biosynthetic pathways related to the source of circular RNAs: (1) whole-exonic-type circRNAs (EcircRNA); (2) circRNAs with introns and exons (EIcirRNA); (3) lasso-type circRNAs composed of introns (ciRNA); and (4) circRNAs produced by cyclization of viral RNA genomes, ribosomal RNAs, small-nuclear RNAs, and transfer RNAs (tricRNA).

CircRNAs are divided into the following four categories: (1) whole-exonic-type circRNAs (EcircRNA); (2) circRNAs with introns and exons (EIcirRNA) (18); (3) lasso-type circRNAs composed of introns (ciRNA); and (4) circRNAs produced by cyclization of viral RNA genomes, ribosomal RNAs, small-nuclear RNAs, and transfer RNAs (tricRNA) (**Figure 1**). Based on their multiple biological mechanisms, circRNAs can retain the ability to either stay inside the nucleus or undergo nuclear export. This selective retention is important for determining the biological roles of different circRNAs and further provides us with research ideas for exploring the potential of circRNAs in the occurrence and development of SZ, DEP, and many other diseases. CircRNA is generally considered to be the result of reverse splicing. This indicates that the downstream 5′ donor site is covalently cyclized with the upstream 3′ receptor site (19). At present, there are three main looping mechanisms of circRNAs.

#### Cable-Tail-Insertion Cyclization Dependent on a Shearing Body

In eukaryotic cells, the formation of circRNAs by exon cable-tail-insertion cyclization (20, 21) is the earliest and most common way of looping, and more than 80% of circRNAs contain exons. Exon cable-tail-insertion cyclization relies on a typical shearing-body mechanism. On the mRNA precursor, the downstream 5′ donor site of the exon is catalyzed to link to the upstream 3′ receptor site by successively assembling small-nuclear ribosomal proteins to form cable-tail-insertion cyclization. Eventually, RNA is formed by further shearing. However, the specific mechanism underlying this process remains unclear.

#### Promotion of circRNA Formation by Cyclic-Acting Elements

Partial introns on both sides of circRNA exons contain reverse complementary sequences that can form RNA double chains side by side at shear sites (22). Finally, two kinds of different circRNAs with or without introns are formed by variable shearing. In general, a nucleotide sequence of 30–40 nt in length can promote the cyclization of circRNAs. Furthermore, exons inside can also compete for RNA pairing and ultimately form different types of circRNAs *via* variable shearing.

#### Regulation of circRNA Formation by RNA-Binding Proteins (RBPs)

In *Drosophila melanogaster*, MBL protein (23) can promote the formation of circRNAs by binding to the introns flanking an exon. Some studies (24) have shown that hundreds of circRNAs are regulated during the process of the epithelial-mesenchymal transition (EMT), and the looping formations of more than one-third of circRNAs are dynamically regulated by the splicing factor, Quaking (QKI). The effect of QKI on circRNA abundance depends on the binding motif of the QKI intron. Critically, the addition of a QKI motif is sufficient to re-induce the formation of circRNA from a linear splicing transcript. In contrast to the effect of QKI protein, high expression of ADAR1 protein (22) can inhibit the formation of circRNAs by destroying the RNA pairing flanking the exon.

Some exon-derived circRNAs are localized in the cytoplasm and contain both ribosome-binding sites and evolutionarily conserved termination codons. Exon-derived circRNAs also share the start codon of the host mRNA, which indicates that hundreds of endogenous circRNAs have translation potential (25), and some of their translation products can carry out functions. Previous studies have confirmed that many (31 out of 132) proteins encoded in ribosomal-related circRNAs (ribo-circRNAs) contain at least one identifiable protein domain. Furthermore, circRNA, such as circMbl, can be translated in a cap-independent manner *in vitro* (26). The same phenomenon is found for circ-ZNF609, which is associated with multiple ribosomes. Circ-ZNF609 translates protein in a splicing-dependent and cap-independent manner. This gene controls the proliferation of primary myoblasts and is the key regulatory factor of their growth (27).

### Molecular Biological Functions of circRNAs

#### Efficient microRNA Sponges

One of the most important molecular biological functions of circRNAs is that they indirectly affect the expression of target gene mRNAs by binding to microRNAs (miRNAs). As an important regulator at the post-transcriptional level, the function of miRNAs is mainly to downregulate or hinder the translation of target genes (mRNAs) by binding to the complementary 3′-end non-coding region located on the target mRNA (28, 29). As miRNA sponges, most circRNAs play an important role in transcriptional and translational regulation of mRNAs by competing for intracellular miRNAs (30, 31).

Among these miRNA sponges, Cdr1as circRNA is highly expressed and stable. It contains more than 70 conserved miRNA response elements (MREs) of miR-7 and one MRE of miR-671, which are then widely combined with AGO2, a key element of gene silencing. Bezzi and colleagues (32) provided evidence to support the functional association among Cdr1as, miR-7, and its target gene. A plasmid expressing Cdr1as was injected into zebrafish embryos to knockdown the expression of miR-7. This effect was partially rescued by injecting miR-7 precursor. At the same time, miR-671 indirectly influenced the expression level of the miR-7 by triggering the degradation of Cdr1as. The interaction between circRNAs and miRNAs plays an important role in tumors, such as breast cancer, liver cancer and cervical cancer (31–35). Han et al. found that circMTO1 was significantly downregulated in human hepatocellular carcinoma (HCC) (36). Silencing of circMTO1, a miRNA-9 sponge, in patients with HCC downregulated the expression of the miRNA9 target gene, p21, thereby promoting the proliferation and invasion of HCC cells. Furthermore, the survival time of HCC cells with low expression of circMTo1 was reduced. This study also found that miRNA9 inhibitors could block the silencing effect of circMTO1. These findings suggest that cycMTO1 may be used as an objective evaluation index of survival prognosis of HCC and may be a potential target for liver cancer treatment. In addition to circMTO1 acting as a sponge for miRNA-9 in HCC, hsa-circRNA-103809 (37) was also found to participate in the pathological mechanism of HCC. It could be used as a sponge for miR-377-3p to increase the expression of its target gene [fibroblast growth factor receptor 1 (FGFR1)] and promoted the proliferation and invasion of HCC cells. Moreover, hsa-circRNA-103809 short-hairpin RNA could act as a tumor inhibitor by downregulating FGFR1 in HCC.

#### Templates for Translating Proteins

N6-methyladenosine (M6A) is the most abundant base modification of RNA. A previous study found (38) that the m6A motif was enriched in circRNAs, and that a single m6A site was sufficient to drive the initiation of circRNA protein translation in human cells. However, this protein translation of circRNA driven by M6A did not function without the following: the activation factor, eIF4G2; the M6A reader, YTHDF 3; enhancement of the methyltransferase, METTL 3/14; inhibition and upregulation of demethylase during heat shock, FTO. Interestingly, further analysis indicated that m6A-driven circRNA translation is widespread.

#### Interactions With RBPs

**Figure 2:**
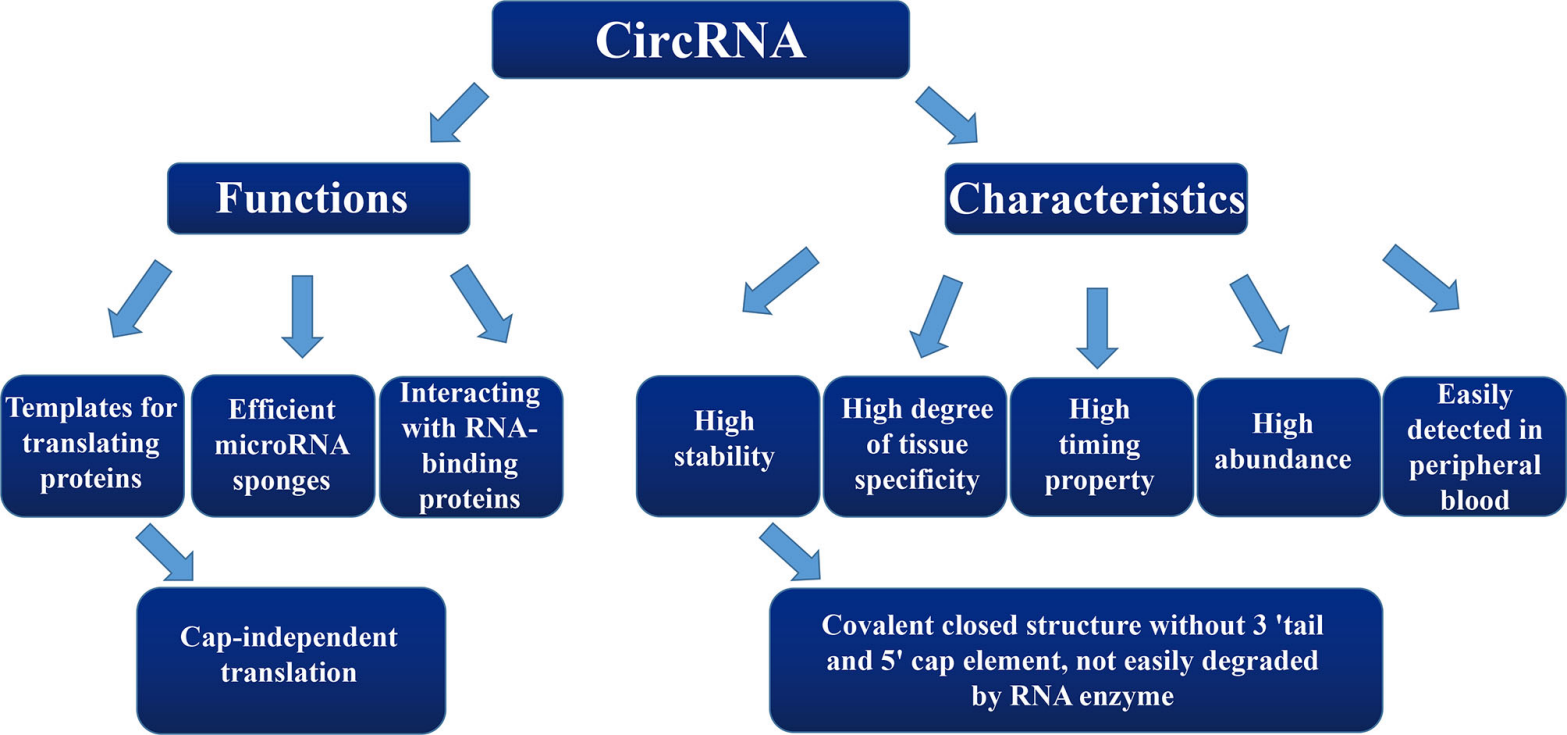
The overview diagram shows multiple biological functions of circular RNAs.

Interactions between circRNAs and RBPs are not only related to their own biological occurrences, but they are also involved in the post-transcriptional regulation of RNAs (39). In general, the cell-cycle protein, cyclin-dependent kinase 2 [also known as cell-division protein kinase 2 (CDK2)], interacts with the cell-cycle proteins A and E to facilitate the cell cycle. However, cyclin-dependent kinase inhibitor 1 (p21) can inhibit these interactions and prevent cell-cycle progression. Du et al. (40) found that circ-Foxo3 was highly expressed in non-cancer cells, forming a ternary complex to inhibit the cell cycle by binding to CDK2 and p21. Additionally, silencing of endogenous circ-Foxo3 promoted cell proliferation. Meanwhile, Zhu et al. (41) showed that circZKSCAN1 exerted its inhibitory effect by competitively binding to the RNA-binding protein (RBP), FMRP. This mechanism blocked the binding of FMRP to the β-catenin-binding protein cell cycle and apoptosis regulator 1 (CCAR1) mRNA, and subsequently inhibited the transcriptional activity of the Wnt signal transduction pathway. In addition, downregulation of the RNA-splicing protein, Quaking5, in HCC tissue caused a decrease of circZKSCAN1. These findings suggest the formation of a Qki5-circZKSCAN1-FMRP-CCAR1-Wnt signaling pathway, which may be useful as a potential therapeutic target for HCC (**Figure 2**).

The above results indicate that circRNAs act not only as miRNA sponges by competing with intracellular miRNA to influence the expression of target genes, but they also act as translational templates for proteins. More importantly, circRNAs lead to altered gene expression outcomes by splicing their corresponding precursor transcripts, which may play an important role in the occurrence and development of various diseases (**Table 1**).

**Table 1:** Molecular biological function of circRNAs

Author	Main findings
Chen and Sarnow (25)	Hundreds of endogenous circRNAs with ribosome binding sites shared the same start codon of their hosting mRNA, which meant they had translation potential.
Bezzi et al. (32)	Cdr1as knocked down the expression of miR-7, and this effect was partially rescued by injecting miR-7 precursor. At the same time, miR-671 indirectly influenced the expression level of miR-7 by triggering the degradation of Cdr1as.
Yang et al. (38)	N6-methyladenosine (m6A) motif was enriched in circRNA, and a single m6A site was sufficient to drive the initiation of circRNA protein translation in human cells.
Han et al. (36)	Silencing of circMTO1, a miRNA-9 sponge, in patients with HCC could downregulate the expression of miRNA9 target gene p21, thereby promoting the proliferation and invasion of HCC cells.
Legnini et al. (27)	Circ-ZNF609 translated protein in a splicing-dependent and cap-independent manner, which controlled the proliferation of primary myoblasts and was the key regulatory factor of their growth.
Zhu et al. (41)	CircZKSCAN1 exerted its inhibitory effect by competitively binding to the RNA binding protein (FMRP). Therefore, the Qki5-circZKSCAN1-FMRP-CCAR1-Wnt signal axis could be used as a potential therapeutic target for HCC treatment.
Zhan et al. (37)	Hsa-circRNA-103809 could be used as a sponge for miR-377-3p to increase the expression of its target gene [fibroblast growth factor receptor 1 (FGFR1)] and promoted the proliferation and invasion of HCC cells.

CircRNAs can act not only as miRNA sponges by competing with intracellular miRNA to influence the expression of its target genes but also as a translation templates for proteins. They can also combine with RBPs directly and realize the regulation of post-transcriptional RNA, which plays an important role in the occurrence and development of diseases.

## CircRNAs and SZ

SZ is one of the most common mental disorders, with a global prevalence of 1%, which brings a heavy burden to affected families and society (42). According to the latest research conducted by Huang et al. (43), the prevalence of SZ is 0.559% in China. Symptoms of SZ are divided into positive, negative, and cognitive categories (44, 45). The diagnosis of SZ is mainly based on symptomatic changes and the clinical experience of psychiatrists. However, these rather subjective measures can cause misdiagnoses and missed diagnoses. Therefore, it is necessary to further explore objective indexes to improve the early diagnosis and prognosis of SZ. Since circRNAs are easily detected in peripheral blood and have a high degree of spatiotemporal tissue specificity and stability (46, 47), these properties provide us with a novel approach to further explore the mechanisms underlying the occurrence and development of SZ.

### Molecular Mechanisms of circRNAs in SZ

As representative antisense nucleic-acid sponge sequences, circRNAs can competitively bind to miRNAs *via* MREs, which negatively influence the regulatory effects of miRNAs at the post-transcriptional level. MiRNA-320a-3p, miRNA-320b, miRNA-181b-5p, 21-5p, 195-5p, 137, 346, 34a-5p, and hsa-miRNA-206 (48–50) have all been confirmed to play important roles in the occurrence and development of SZ. Wei et al. (51) found that eight miRNAs were upregulated in patients with SZ, as compared to corresponding levels in control participants. Through quantitative reverse-transcription polymerase chain reaction (qRT-PCR) assays and a follow-up study on 400 patients with SZ who received regular atypical antipsychotic treatment for 12 months, this study ultimately showed that miR-130b and miR-193a-3p were upregulated in SZ, and the possibility of time-dependent changes were ruled out. Hence, miR-130b and miR-193a-3p may be used as state-independent biomarkers for SZ. A recent study (52) has confirmed that the expression of the network axis composed of miR-30a-5p, its transcription factor, EGR1 and target gene, NEUROD1, changed with the disease state in the patients with SZ before and after treatment. Further receiver operating characteristic (ROC) curve analysis revealed that compared with the single miR-30a-5p, the EGR1-miR-30a-5p-NEUROD1 molecular regulatory axis had higher diagnostic value for predicting SZ. Hence, these findings suggest a possibility for the miRNA-TF-gene regulatory network/axis as a novel diagnostic marker for SZ.

Due to their circular structure, circRNAs are highly conserved across species. Some studies have confirmed that circRNAs are highly expressed in the brain and are differentially expressed in distinct tissues and disease states (53, 54). Moreover, circRNAs exhibit dynamic expression levels during neural development, including neural differentiation and maturation. For instance, circRNAs are upregulated during neuronal differentiation, and compared with their mRNA homologues, they generally exhibit differential expression (55–57). SZ often occurs in late adolescence and early adulthood. Previous studies have suggested that excessive pruning of synapses in the brain during growth and development may contribute to the pathogenesis of SZ (58).

Some studies have suggested synaptic localization of circRNAs. Wolf et al. (56) found that circRNAs in the nervous system were generally upregulated compared to those in the thyroid, liver, and muscle, and that expression varied among different brain regions. Especially in the cerebellum, the expression ratio of circular to linear RNAs was significantly increased, which correlated with a larger number of neurons in the cerebellum compared to that in other brain regions. Furthermore, by measuring high-purity synaptosomal components, this study found that brain-expressed circRNAs had a strong enriching effect at synapses. Another study found that circStau2a was mainly located at synapses, whereas its linear transcript, mRNAStau2, was almost completely localized to the cytoplasm (56). Similarly, circRNA derived from the known neuronal differentiation regulator, RMST, exhibits a high synaptic enrichment rate (59). It has also been found that during the development of the hippocampus, upregulated circRNAs are produced by the gene locus that simultaneously encodes proteins rich in synapse-related functions. Conversely, no enrichment of encoding of any other functional class of proteins was found in the gene locus that produced a downregulated dynamic expression pattern of circRNAs (29, 60, 61). In summary, the expression levels of circRNAs are regulated by neuronal development. Many circRNAs also change the structures of neurons, which are largely independent of the function of their linear transcripts.

### Expression of circRNAs in Postmortem SZ Brains

Our literature review showed that the most extensive studies of circRNAs and SZ have involved the analysis of the expression profiles of circRNAs based on control studies of patients with SZ and the healthy controls matched to them. Mahmoudi et al. used circRNA enrichment sequencing to analyze the expression profiles of circRNAs in the cerebral cortex (BA46) of 35 postmortem patients with SZ as well as those of healthy controls (62). They found that more than 95,000 types of circRNAs in the human dorsolateral prefrontal cortex (DLPFC) showed significant diversity, and that half of them had not previously been reported, such as circHomer1, circKlhl2, circMpped1, and circNell2 (63, 64). A differential analysis of these circRNAs revealed that 390 circRNAs were downregulated and 240 were completely deleted in patients with SZ. In addition to the reduced overall level of gene expression in circRNAs, the total numbers of unique circRNAs found in SZ were also reduced compared to those in heathy controls, and there was a large overlap between them. For instance, circNEll2, which is upregulated after synaptic plasticity induction, was downregulated in patients with SZ. The researchers also explored the correlation between differentially expressed circRNAs and their linear isoforms. Surprisingly, the expression levels of more than half of the circRNAs showed an inverse relationship with those of their linear RNAs, suggesting that both forms of transcripts may sometimes antagonize each other’s biosynthesis. Zimmerman et al. (65) also discovered circHomer1a, which is highly expressed and neuron-enriched in the frontal cortex. Interestingly, in the prefrontal cortex (PFC) and induced pluripotent stem cell-derived neuronal cultures in patients with SZ and BD, circHomer1a was found to be significantly downregulated. Additionally, changes in circHomer1a expression in the DLPFC and orbitofrontal cortex (OFC) were positively correlated with the age of onset of SZ. In addition, circHomer1a was found to interact with RNA-binding protein, HuD, through animal-level verification, which was involved in regulating synaptic gene expression and cognitive flexibility. These findings are of great significance for exploring the molecular mechanisms underlying the pathogenesis of mental disorders.

The above results suggest that the overall expression levels of circRNAs are downregulated in patients with SZ, while there are still some unique and significantly differentially expressed circRNAs that differ from this overall trend. These circRNAs may play an important role in the clinical phenotypes of SZ by regulating corresponding cellular metabolic pathways underlying this mental disorder. Furthermore, there is a strong possibility of abnormal regulation of circRNAs in other mental illnesses and behavioral disorders.

### Expression of circRNAs in Peripheral Blood of Patients With SZ

CircRNA plays a particularly important role in the regulation of gene expression in SZ (62, 66, 67). Previous studies (68) have identified that differentially expressed circRNAs from peripheral blood perhaps contribute on the development of the diagnosis and treatment of SZ.

A case-control study carried out by Yao and colleagues (69) screened nine kinds of significantly expressed circRNAs. Further verification by qRT-PCR revealed that three circRNAs were downregulated and two circRNAs were upregulated in the SZ group. The expression levels of circRNAs in patients with SZ after 4 and 8 weeks of conventional antipsychotic treatment were then re-quantified to see if there was any change. ROC curve analysis showed that three circRNAs (hsa circRNA 103704, hsa circRNA 101836, and hsa circRNA 104597) were of diagnostic significance, with a sensitivity of 87.25% and specificity of 85.44%. In particular, the sensitivity and specificity of hsa circRNA 104597 were 84.31% and 86.41%, respectively, indicating that it may be useful in the diagnostic and therapeutic evaluation of SZ.

The above results suggest that circRNAs may provide novel ideas for exploring objective biomarkers for SZ diagnosis. However, there has only been one study on this topic, which had some limitations. For instance, the sample size of screening differentially expressed circRNAs was relatively small. Since patients with SZ had been treated with standardized antipsychotic drugs for a relatively short period of time, changes in the expression level of circRNAs before and after treatment may also be due to their own time-dependent changes. Furthermore, only one type of circRNA was found to may represent a novel objective diagnostic value for SZ. Finally, there are still many significantly expressed circRNAs related to SZ that require further exploration (69–71).

## CircRNAs and Depression

Depression (DEP) is one of the most common mental disorders in humans. The World Health Organization (WHO) has reported that more than 800 million people worldwide suffer from DEP (72, 73). Approximately 40–60% of patients with DEP show suicidal ideation or behavior, and the suicide rate has been reported to be as high as 15% (74, 75). The disease burden of DEP is increasing year by year. In 1990, DEP ranked fifth in terms of its global burden as a disease. However, DEP is expected to become the top disease burden in China by 2030 (76). The process of diagnosing this disease is similar to that for SZ and, as with SZ, it can easily lead to misdiagnoses and missed diagnoses. Therefore, it is necessary to further explore the pathological mechanisms and specific molecular markers related to DEP to improve the early diagnosis and prognosis of DEP.

### Expression of circRNAs in Peripheral Blood in Depression

Cui and colleagues randomly selected peripheral blood mononuclear cells (PBMCs) from five patients with DEP and five healthy controls to analyze the expression profile of circRNAs. First, 15 differentially expressed circRNAs (including four upregulated and 11 downregulated circRNAs) were screened (77). Then, four significantly differentially expressed circRNAs were identified through verification in 100 DEP patients, 103 healthy controls, and 30 randomly selected DEP patients after 4 and 8 weeks of antidepressant therapy. However, only hsa-circRNA-103636 showed significant changes in DEP patients after 8 weeks of antidepressant treatment. Further ROC curve analysis showed that the sensitivity and specificity of this gene were 0.73 and 0.65, respectively. Therefore, hsa-circRNA-103636 may have potential diagnostic value of DEP.

Many studies have shown that type-2 diabetes (T2DM) is closely related to the occurrence and development of DEP (78, 79). Tian et al. collected venous plasma from five patients with T2DM and five cases of T2DM with DEP to determine the expression profiles of ncRNAs (including lncRNAs, circRNAs, and mRNAs) in order to discover specific molecular markers associated with DEP. The results of screening identified 28 lncRNAs, 107 circRNAs (including 75 upregulated and 32 downregulated circRNAs), and 89 mRNAs in the differential expression profiles of patients with DEP (80). Compared with that in the T2DM group, circRNA-TFRC had a significantly higher expression level in the DEP group. This circRNA is related to insulin resistance, which indicates that patients with DEP may have more severe insulin resistance symptoms. Some studies (81) have found that the expression level of TFRC is associated with mental disorders, suggesting that TFRC may be useful as a molecular target for studying the pathogenesis of DEP. Another upregulated circRNA-TNIK [Traf 2- and Nck-interacting kinase (TNIK)] is also associated with DEP. TNIK is a serine/threonine kinase that exhibits a high expression level in the brain, which has a good effect on the development and synaptic transmission of dendritic cells. A previous study (80) found that compared with a T2DM group, the expression level of TNIK was significantly increased in the DEP group. This suggests that the function of TNIK merits further investigation.

Jiang and colleagues (82) used microarrays to analyze plasma samples from seven patients with T2DM and seven patients with T2DM and DEP. Compared with the T2DM group, 183 circRNAs were upregulated and 64 circRNAs were downregulated in T2DM patients with DEP. Among them, the upregulated gene, DCP2 (hsa-circRNA-001520), may be used as a type of de-capping enzyme and plays an important role in neuronal development and mental retardation by degrading mRNAs. Another downregulated gene, CSGALNACT1 (hsa-circRNA-001781), is associated with an antidepressant response (83). In addition, Zhang et al. demonstrated that circDYM was also significantly decreased in MDD patients and two depressive-like mouse models [induced *via* chronic unpredictable pressure (CUS) and lipopolysaccharide (LPS)]. Recovery of circDYM levels significantly ameliorated CUS- or LPS-induced depressive-like behavior, which suggests that circDYM may be a novel therapeutic target for DEP (84).

According to the target genes of circRNAs related to DEP predicted by TargetScan and Miranda, 18 miRNAs and 529 mRNAs were found to interact with 4 circRNAs. Interestingly, hsa-miR-761 and hsa-miR-298 are common targets for hsa-circRNA-003251, hsa-circRNA-005019, and hsa-circRNA-015115. A previous study found that hsa-circRNA-003251 and hsa-circRNA-015115 may play important roles in the circRNA-miRNA-mRNA network associated with DEP by acting as an hsa-miR-761 sponge. Hsa-miR-761 (85, 86) has been shown to be involved in the regulation of mitochondrial networks and to promote the generation of learning and memory. Furthermore, overexpression of hsa-miR-761 inhibits the p38-MAPK signaling pathway (87). Therefore, hsa-miR-761 may participate in the pathological mechanisms of DEP. In addition, KEGG pathway analysis predicted that upregulation of hsa-circRNA-003251, hsa-circRNA-015115, hsa-circRNA-100918, and hsa-circRNA-001520 might be involved in the Wnt signaling pathway implicated in thyroid hormones. Another study showed that thyroid hormones affected mood and promoted signal pathways in the brain (88). These findings suggest that the occurrence and development of DEP might also be related to changes and metabolic disorders involving thyroid hormones (**Table 2**).

**Table 2:** Expression of circRNAs in schizophrenia and depression

Author	Main findings
Ng et al. (59)	CircRNA derived from the known neuronal differentiation regulator RMST had a very high synaptic enrichment rate.
You et al. (60)	During the development of the hippocampus, the upregulated circRNA is produced by the gene locus that simultaneously encodes protein rich in synapse-related functions.
Hanan et al. (57)	CircRNA in the nervous system is generally upregulated compared to the thyroid, liver, and muscle. Especially in the cerebellum, the expression ratio of circular and linear RNA is significantly increased, and the brain-expressed circRNAs, such as circStau2a, have a strong enrichment effect on synaptic-neurons.
Mahmoudi et al. (62)	A total of 390 circRNAs, such as circNEll2, were down-regulated and 240 were completely deleted in patients with SZ. In addition to the reduced overall level of circRNA expression, the total numbers of unique circRNAs found in SZ were also reduced. More than half of circRNAs might sometimes antagonize linear RNA biosynthesis.
Yao et al. (68)	Three circRNAs (hsa circRNA 103704, hsa circRNA 101836, and hsa circRNA 104597) were of diagnostic significance, with a sensitivity of 87.25% and a specificity of 85.44%. In particular, the sensitivity and specificity of hsa circRNA 104597 were 84.31% and 86.41%, respectively, indicating that it might be a new potential biomarker for the diagnostic and therapeutic evaluation of SZ.
Abasolo et al. (81)	The expression level of TFRC is associated with mental disorders, suggesting that TERC could use as a molecular target for studying the pathogenesis of DEP.
Cui et al. (77)	Fifteen differentially expressed circRNAs (including 4 upregulated and 11 downregulated circRNAs) were screened out by a case-control study. However, only hsa-circRNA-103636 showed significant changes in depressed patients after 8 weeks of antidepressant treatment, indicating that it could be used as a new potential biomarker for the diagnosis and treatment of DEP.
Jiang et al. (82)	Compared with the T2DM group, 183 circRNAs were upregulated and 64 circRNAs were downregulated in T2DM patients with DEP. Hsa-circRNA-003251 and hsa-circRNA-015115 might play important roles in the circRNA-miRNA-mRNA network associated with DEP by acting as hsa-miR-761 sponges. In addition to the above two RNAs, hsa-circRNA-100918 and hsa-circRNA-001520 might be involved in the Wnt signaling pathway of thyroid hormone.
An (80)	Compared with the T2DM group, circRNA-TFRC had a higher expression level and circRNA-TNIK was significantly increased in the DEP group. TFRC was related to insulin resistance, which indicated that patients with DEP had more severe insulin resistance symptoms. TNIK had a good effect on the development and synaptic transmission of dendritic cells.

CircRNAs have specific expression profiles in brain regions and peripheral venous blood. Based on these, researchers used case-control studies to explore significantly expressed circRNAs in schizophrenia and depression in order to find the molecular bases of these two diseases.

Based on the above findings, circRNA is a kind of biological signal that is measurable in physiological, pathological, and therapeutic process and perhaps contribute on predicting risk assessments, early diagnoses, and monitoring of responses in patients with SZ or DEP. At present, although some studies on the specific expression levels of circRNAs in various diseases have emerged, most of them have been at the level of expression profiles. Therefore, further studies are needed to better elucidate the precise associations between circRNAs and clinical symptoms in various diseases.

## Limitations and Future Research

At present, an increasing number of studies on circRNAs related to SZ and DEP are emerging. However, there are still many questions to be answered, such as the following: whether changes in a single circRNA or among a group of circRNAs are specific to SZ and DEP; whether levels of circRNAs change with age; whether the expression of circRNAs are affected by smoking or drug abuse; and whether the circRNAs in cellular compartments have any differences in terms of their molecular bases of SZ and DEP (e.g., extra-vesicular circRNAs). These limitations indicate fruitful directions for future studies that may help elucidate the potential diagnostic and therapeutic value of circRNAs for SZ and DEP.

## Author Contributions

YX designed and supervised the study. ZL and SL drafted the manuscript. XL, WZ, and JL collected some literature.

## Funding

This work was supported by the National Natural Science Foundation of China, (81701326 and 81971601), the Support Program of the Youth Sanjin Scholars, and the 136 Medical Rejuvenation Project of Shanxi Province.

## Conflict of Interest

The authors declare that the research was conducted in the absence of any commercial or financial relationships that could be construed as a potential conflict of interest.
